# Gait Kinematics and Asymmetries Affecting Fall Risk in People with Chronic Stroke: A Retrospective Study

**DOI:** 10.3390/biomechanics2030035

**Published:** 2022-09-02

**Authors:** Shuaijie Wang, Tanvi Bhatt

**Affiliations:** Department of Physical Therapy, University of Illinois at Chicago, Chicago, IL 60612, USA

**Keywords:** slip, trip, environmental falls, fall prevention, gait pattern, hemiparesis

## Abstract

Stroke survivors are at a relatively higher risk of falling than their healthy counterparts. To identify the key gait characteristics affecting fall risk in this population, this study analyzed the gait kinematics and gait asymmetries for 36 community-dwelling people with chronic stroke (PwCS). According to their fall history in the last 12 months, they were divided into a fall group (*n* = 21) and non-fall group (*n* = 15), and then the gait kinematics (step length, stride length, stance time, swing time, trunk angle, and segment angles for lower limbs) and their asymmetries (symmetry ratio and symmetry index) were compared between these two groups. To investigate the relationship between fall types and gait characteristics, these variables were also compared between 11 slip-fallers and non-fallers, as well as between 7 trip-fallers and non-fallers. Our results indicated that the fallers showed smaller trunk and thigh angle, larger shank angle, and higher gait asymmetries (trunk and foot). Such changes in gait pattern could also be found in the trip-fallers, except the trunk angle. Additionally, the trip-fallers also showed a shorter step length, shorter stride length, shorter swing time, larger foot angle on the paretic side, and higher asymmetries in shank angle and step length, while the slip-fallers only showed changes in trunk angle and thigh angle and higher asymmetries in step length and foot angle compared to the non-fall group. Our results indicated that improper or pathological gait patterns (i.e., smaller thigh angle or higher foot asymmetry) increases the risk of falling in PwCS, and different fall types are associated with different gait characteristics. Our findings would be helpful for the development of fall risk assessment methods that are based on kinematic gait measurements. Implementation of objective fall risk assessments in PwCS has the potential to reduce fall-related injuries, leading to a reduction in associated hospital costs.

## 1. Introduction

People with stroke commonly experience falls [1,2] and have higher fall rates than older adults in general [3,4]. Over 40% of community-dwelling people with chronic stroke (PwCS) experience detrimental falls during walking each year [5,6], with consequences of such falls including traumatic head injuries, a four-fold increase in the risk of hip fracture, and even mortality [5,6]. To reduce the impact of falls on PwCS, it is important to develop accurate methods of fall risk identification that can identify stroke patients with a high risk of falling. Accurate fall risk identification could thus be used to design and implement targeted fall prevention paradigms to reduce fall incidence in PwCS with high fall risk.

Several studies have identified fall risk factors in PwCS, primarily based on clinical measures (i.e., motor impairment, sensory impairment, and performance on activities of daily living) and gait characteristics (i.e., spatio-temporal parameters) [7]. Assessing fall risk through gait analysis has become increasingly popular in clinical practice [8–10] as gait patterns can easily be quantified using portable wearable sensors (accelerometers and gyroscopes) [11] and can be used to assess physical function, muscle power, and dynamic balance in clinical and home environments [12–15]. Previous studies have reported altered gait patterns to be associated with impaired motor control and decreased lower limb muscle strength in older adults and patients with mild cognitive impairment [16–20], which are factors that can affect the ability to maintain stability (i.e., control the center of mass) and increase fall risk. Fall-risk evaluation models developed using spatial (step length, stride length, gait speed) and temporal (swing time, stance time) gait parameters during walking show moderate to high accuracy for fall risk prediction [21–24].

One predictor of fall risk in PwCS is gait asymmetry [8,25–28], which is defined by differences in the bilateral behavior of the lower limbs during regular walking [25]. Gait asymmetries commonly appear as a result of stroke [29] and are associated with impaired balance [28] and increased energy cost [30] during locomotion. A previous study proposed that the level of asymmetries in gait parameters might be more relevant to the compensatory mechanisms (to improve balance recovery) used during locomotion than the gait parameters themselves [31]. Additionally, the asymmetries of swing time and stance time are significantly larger in fallers than non-fallers, suggesting that gait asymmetry is an important factor for fall prediction in PwCS [28].

Although previous studies have analyzed gait parameters and their asymmetries as predictors of fall risk, these studies have primarily examined spatio-temporal factors rather than joint/segment kinematic patterns between fallers and non-fallers in PwCS. As spatiotemporal gait parameters are determined by the coordination of lower limb segments [32], the analysis of lower extremity angles and their asymmetries could provide additional insight into how lower limb segment coordination is associated with fall risk. Previous studies in healthy older adults have revealed that larger hip flexion of the leading limb can increase the risk of slip-induced fall [24], and fallers have a larger knee flexion and ankle dorsiflexion during regular walking [33]. It is possible that segment/joint kinematics in PwCS could also differentiate fallers and non-fallers, however there is very limited research on this topic.

Additionally, previous studies have reported that individuals who experience different types of falls have different gait patterns, and further, different spatio-temporal gait parameters were found to exist among individuals who fall in different directions or different environments [34–37]. Therefore, it is possible that gait kinematics and asymmetries could not only predict fall risk but also indicate what types of fall an individual is more likely to experience. However, previous studies have not investigated fall risk assessment for specific types of falls (i.e., only for overall number of falls), which could result in poor prediction accuracy and even heterogeneous conclusions. Considering that slips and trips are the most frequent (over 50%) self-reported causes of outdoor falls among older adults, this study also aimed to investigate the gait kinematics and asymmetries associated with fall risk for a specific type of fall (slips and trips).

Thus, the primary purpose of this study was to investigate the key gait kinematics and gait asymmetries affecting fall risk in PwCS. We hypothesized that fallers would show significantly different gait patterns compared to non-fallers, reflected by different gait kinematics and higher gait asymmetries. Additionally, we explored whether gait kinematics and asymmetries were related to fall risk for a specific type of fall (slip or trip).

## 2. Method

### 2.1. Participants

Thirty-six community-dwelling PwCS with >6 months post-cortical stroke and with Chedoke–McMaster Stroke Assessment (CMSA) ≤5, confirmed by their physician were included in this study. All participants were able to ambulate independently without an assistive device for over 30 min. They firstly responded to a general health questionnaire by screening to ascertain their health status. Specifically, participants with broken bones and surgery within 6 months, known history of peripheral nerve injury in the lower legs, history of cardiovascular or pulmonary complications, or with pacemakers and history of metabolic (endocrine, hepatic) or renal dysfunction were excluded. Next, they were screened via clinical measures before their training session, and they were excluded if they had cognitive impairments (score of ≤26/30 on Montreal Cognitive Assessment Scale), speech impairments (aphasia score of ≥71/100 on Mississippi Aphasia Scale), low bone density (T score < *−*2 on the heel ultrasound), or the presence of neurological, musculoskeletal, or cardiovascular impairments during the in-person screening. Our inclusion and exclusion criteria could ensure that the abnormal gait patterns in the participants were mainly attributed to stroke-related sensorimotor and balance impairments. The study was approved by the Institutional Review Board at the University of Illinois at Chicago, and all participants provided written informed consent.

### 2.2. Experimental Setup

Participants were instructed to walk on a 7 m walkway at their preferred speed and in their preferred manner for at least 10 trials to acquaint themselves with walking in the new laboratory environment. During all trials, participants were equipped with a full-body safety harness which was connected to an overhead trolley on a track above the walkway. The harness allowed participants to walk freely while providing protection by preventing the body from coming into contact with the floor surface. Kinematics from a modified Helen Hayes full-body marker set (30 retro-reflective markers) were recorded by an eight-camera motion capture system (Motion Analysis Corporation, Santa Rosa, CA, USA). Kinematic data was sampled at 120 Hz and synchronized with the force data at 600 Hz, which was collected by force plates (AMTI, Newton, MA, USA) installed beneath the walkway. The last three walking trials were analyzed in this study.

### 2.3. Retrospective Fall History

Fall status was determined using a self-report questionnaire, which asked whether the participant had fallen in the 12 months prior to the experiment, how many falls they experienced in the last 12 months, and what caused each fall. A fall was defined as any event that resulted in the participant unintentionally coming into contact with the ground or floor. Based on fall history, participants were divided into the fall group if they fell at least once in the previous year (*n* = 21) or non-fall group if they did not fall in the previous year (*n* = 15). Out of the 21 fallers, 11 of them experienced at least one slip-induced fall, 7 of them experienced at least one trip-induced fall, and 2 of them were recurrent fallers who experienced both slip- and trip-induced falls. Other causes of falls included activities of daily living (i.e., bathing, homemaking, cleaning), fainting, dizziness, and unsure causes.

### 2.4. Data Analysis

A custom MATLAB program (The MathWorks, Natick, MA, USA) was used to compute gait kinematics and the corresponding gait symmetry. These parameters were calculated using the 3-D motion data (XYZ coordinates of markers), which were filtered with a fourth-order low-pass Butterworth filter with a cut-off frequency of 10 Hz. As only the variables in the sagittal plane were calculated, the Y coordinate (medial–lateral) was not used.

**Figure 1.**
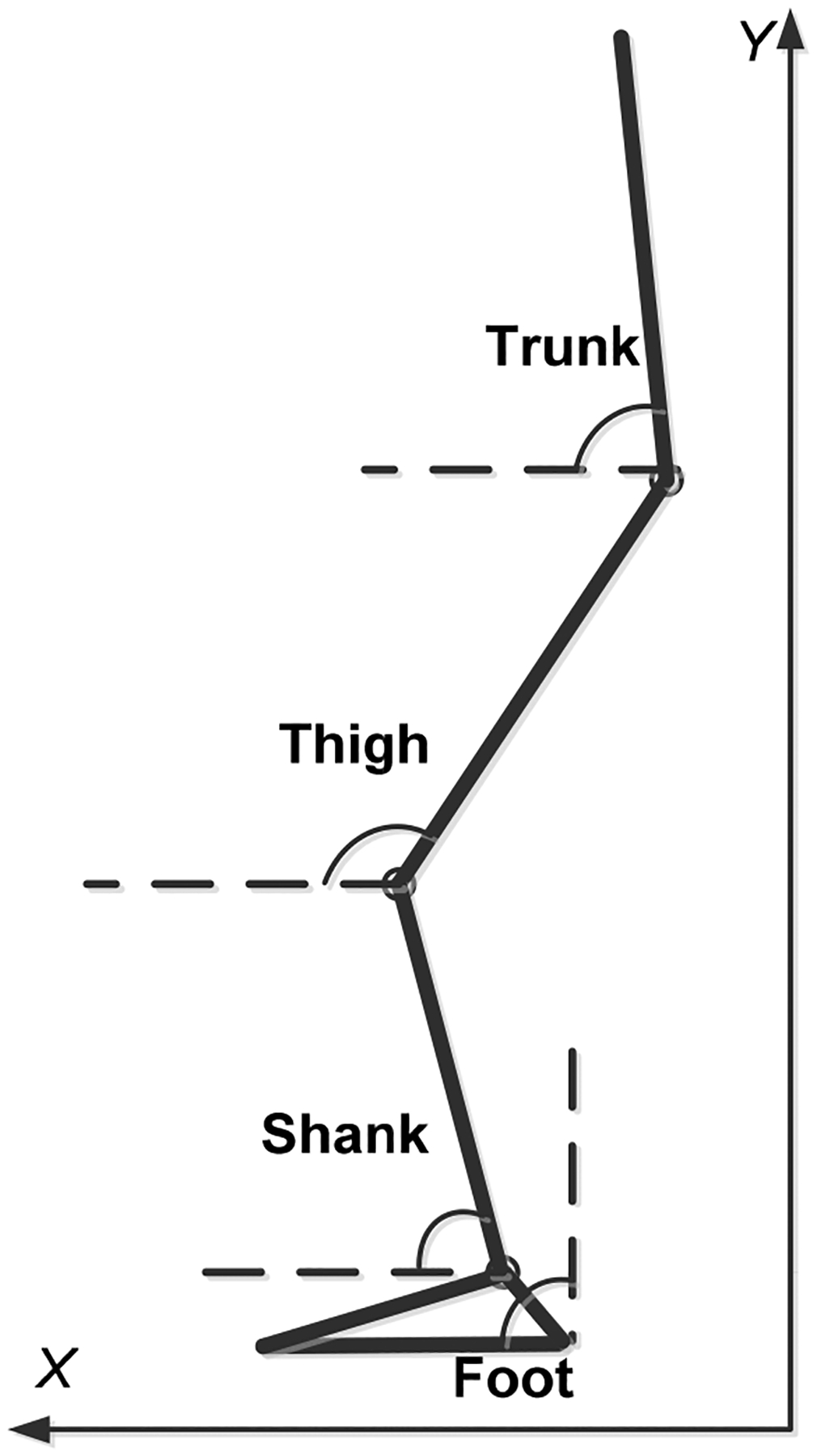
Schematic of the human body model in the sagittal plane. Trunk, thigh, and shank angles were calculated relative to the horizontal plane, and the foot angle was calculated relative to the vertical plane.

The gait kinematics included step length, stride length, stance time, swing time, trunk angle, and the segment angles of the stepping limb (thigh angle, shank angle, and foot angle) at paretic foot touchdown (PTD) and non-paretic foot touchdown (NPTD). Step length was calculated as the heel distance between both feet in anteroposterior (AP) direction at PTD and NPTD, and stride length was the travel distance of the heel marker in one gait cycle in AP direction. Stance time was the duration from the touchdown of the leading foot to its liftoff, and swing time was the duration from the liftoff of the trailing foot to its liftoff. All the segment angles in the sagittal plane were computed based on their proximal and distal ends (Figure 1). The trunk, thigh, and shank angles were calculated relative to the horizontal plane, while the foot angle was calculated relative to vertical plane to avoid a near-zero value. The gait kinematics were calculated using two gait cycles for each trial, and the average value of the two cycles were used in this study. To reveal the overall stability, center of mass (COM) position was also calculated as the distance between the projected COM location (estimated using a 13-segment model) and the posterior edge of base of support (BOS), and then normalized by the length of BOS (distance from trailing from trailing heel to leading toe). The crucial time events included PTD, NPTD, and the following liftoff, which were detected from force plate data for each trial.

Gait symmetries for each parameter were calculated based on the calculated gait kinematics for paretic side and non-paretic side in two different ways:

(1) $$\text{Symmetry}\;\text{Ratio}\;(\text{SR})=x_{p}/x_{\text{np}}$$

(2) $$\text{Symmetry}\;\text{index}\;(\text{SI})=|x_{p}-x_{\text{np}}|/(x_{p}+x_{\text{np}})/2$$

where *x*_*p*_ and *x*_*np*_ are the value of gait kinematics of the paretic and non-paretic limb. A SR value close to 1 indicates high symmetry between the paretic and non-paretic side, while an SI value close to 0 indicates high symmetry.

### 2.5. Statistical Analysis

Anthropometric measures, including height, weight, age, gender, foot length, foot-to-hip height, leg length (ground to hip height), and femur length, were firstly compared between fall (21 fallers) and non-fall groups (15 non-fallers) using independent test and chi-square test (for gender). To test the difference in gait parameters and symmetries between fall and non-fall groups, 2-way ANOVA was conducted to examine the group effect (*n* = 2), trial effect (*n* = 3), and group by trial interaction. Post hoc paired tests were conducted if there were any significant group effects, trial effects and/or interactions between trial and group. Similarly, 2-way ANOVA was also conducted to detect the difference between slip-fall group (*n* = 11) and non-fall group as well as between trip-fall group (*n* = 7) and non-fall group. Post hoc paired tests were conducted if there were any significant group effects, trial effects and/or interactions between them. Further, a power analysis was conducted for the key factors with significant group effect, effect size (Cohen’s d), and power, and the sample size required for power >80% were calculated. The statistical significance for 2-way ANOVA and the post hoc paired tests was denoted for *p* < 0.05, and statistical analyses were performed using SPSS.

## 3. Results

**Figure 2.**
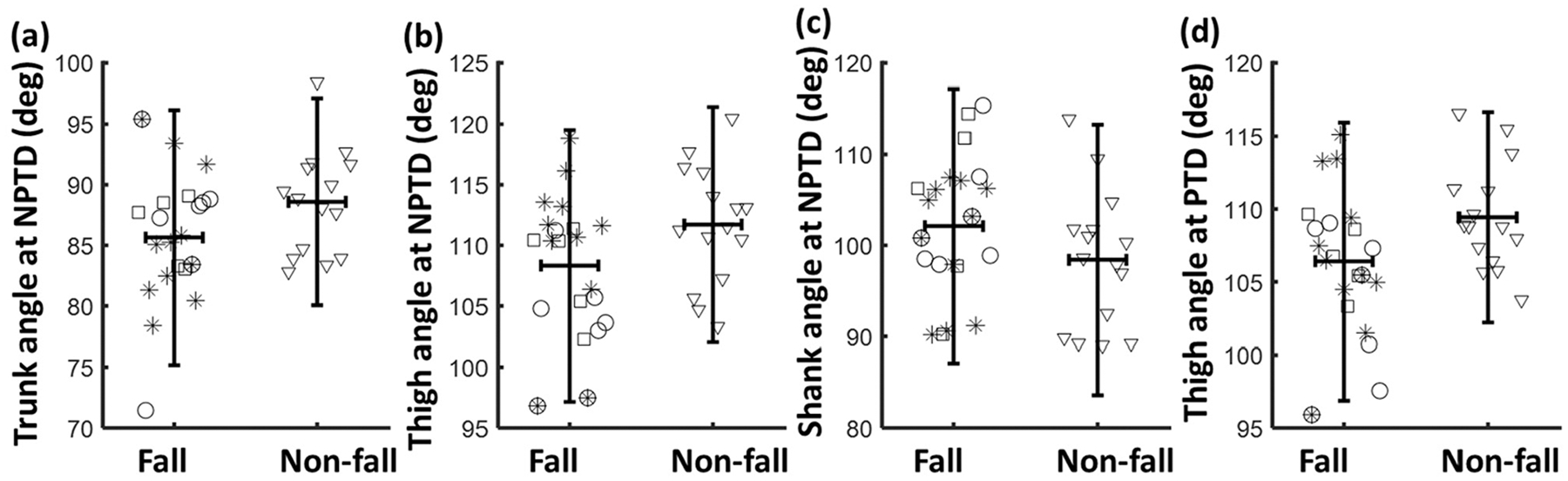
The distribution of gait kinematics between fallers and non-fallers for (**a**) trunk angle at non-paretic foot touchdown (NPTD), (**b**) thing angle at NPTD, (**c**) shank angle at NPTD, and (**d**) thigh angle at paretic foot touchdown (PTD). There were group differences for all these kinematics; the horizontal line denotes the mean value, and the vertical line denotes the 95% confidence interval (mean ± 1.96 SD). The circle marker denotes trip-falls, star marker denotes slip-falls, square marker denotes other types of falls, and triangle denotes non-falls.

No differences were found in the anthropometric measures between the fallers and non-fallers (Table 1). For all spatio-temporal gait parameters (step length, stride length, stance time, and swing time), no difference was found between fall and non-fall groups at both NPTD and PTD (*p* > 0.05 for all, Table 2 and Figure 2), while a majority of the segment angles showed a significant group effect except the foot angle. Specifically, trunk angle (*p* < 0.01) and thigh angle (*p* < 0.01) at NPTD were significantly smaller in fallers compared to the non-fallers; shank angle (*p* < 0.05) at NPTD was significantly larger in the fallers; and the thigh angle at PTD was significantly smaller in the fallers than the non-fallers (*p* < 0.01).

**Figure 3.**
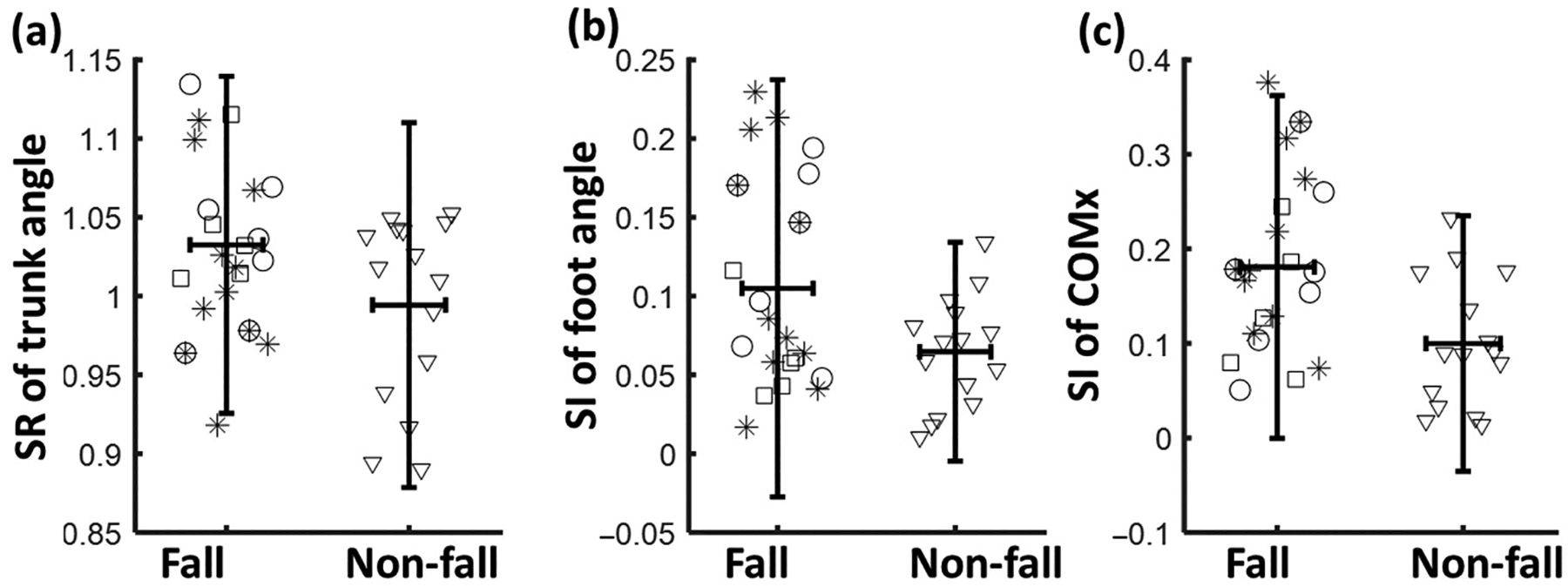
The distribution of gait asymmetries between fallers and non-fallers for (**a**) symmetry ratio (SR) of trunk angle, (**b**) symmetry index (SI) of foot angle, and (**c**) SI of COM position (COMx). There were group differences for all these variables; the horizontal line denotes the mean value, and the vertical line denotes the 95% confidence interval (mean ± 1.96 SD). The circle marker denotes trip-falls, star marker denotes slip-falls, square marker denotes other types of falls, and triangle denotes non-falls.

For gait symmetries, only the SR of trunk angle (*p* < 0.01, Table 3 and Figure 3) and the SI of foot angle (*p* < 0.01) showed a significant difference between fall and non-fall groups, indicating a higher asymmetry in fallers.

The slip-fall group showed similar gait kinematics to the non-fall group, and only the trunk angle at NPTD and thigh angle at PTD were smaller (indicating larger trunk flexion and smaller hip flexion) than the non-fall group (*p* < 0.05 for both, Table 2). For gait symmetry, the slip-fall group showed a lower symmetry of step length (*p* < 0.05), foot angle (*p* < 0.001), and COM position (*p* < 0.001, Table 3) compared to the non-fall group.

A significant group effect was found for a few gait kinematics in the trip-fall group, including the stride length (*p* < 0.05, Table 2), thigh angle (*p* < 0.001) and shank angle (*p* < 0.05) at NPTD, and the step length (*p* < 0.05), stride length (*p* < 0.05), swing time (*p* < 0.01), thigh angle (*p* < 0.001) and foot angle (*p* < 0.05) at PTD. For gait symmetry, the trip-fallers showed a lower symmetry of step length (SI, *p* < 0.05, Table 3), trunk angle (SR, *p* < 0.01), shank angle (SR, *p* < 0.05), foot angle (SI, *p* < 0.001), and COM position (SI, *p* < 0.01) compared to the non-fall group. Power analysis showed that the key factors affecting fall risk showed a moderate to high effect size (0.5 to 0.8), resulting in a power over 70%.

## 4. Discussion

Our results indicated that people with chronic stroke who had a fall history showed, on average, different segment angles (trunk, thigh, and shank) and higher gait asymmetries (trunk and foot). These results are consistent with our hypothesis that fall risk would affect both gait kinematics and gait asymmetries in PwCS. For the two subgroups, trip-fallers also showed these same differences compared to the non-fall group, except difference in trunk angle. Further, trip-fallers had a shorter average step length, stride length and swing time, smaller average thigh angle (less hip flexion), and larger average foot angle (less dorsiflexion) on the paretic side, along with higher asymmetries in trunk angle, shank angle, step length, and foot angle. On the other hand, the slip-fallers only showed a smaller average trunk angle at NPTD and smaller average thigh angle at PTD. The slip-fall group also showed higher asymmetries in step length and foot angle compared to the non-fall group. Such findings indicate that different fall types are associated with different gait characteristics.

We found no difference in spatio-temporal gait parameters (step length, stride length, stance time, and swing time) between the fall group and non-fall group. Such results were consistent with previous findings that there was no difference in stride time and step length between faller and non-faller PwCS [8,38]. However, another study reported that non-fallers have larger step length for both paretic and non-paretic sides than fallers [39]. It is possible that the findings of the previous study were inconsistent with our results because different durations were used to classify fall status; fallers and non-fallers were classified based on 6-month falls in the previous study, whereas we used 12-month falls. It is possible that the difference in the period of data collection would significantly affect the fall rate and identification of fallers versus non fallers [40]. For example, the probability of a person to experience a fall in 6 months is lower than that over 12 months, hence using a 6-month window could have missed many of those who could have had a fall over 12 months. Thus, even for the same set of participants, classification methods based on different periods would lead to different sample sizes for each group, which in turn could have affected the kinematic outcomes resulting differences between the study by Punt et al. [39] and ours. Another explanation for the difference between the two studies could be the differences in the inclusion criteria between studies. We only included PwCS with lower level of independence (CSMA ≤ 5) in our study, whereas this previous study did not exclude PwCS based on their level of independence. As gait asymmetry was found to be significantly correlated to CSMA leg (r = −0.767) and foot (r = −0.759) scores [41], the difference in population might also contribute to the inconsistency. Although we found no difference in step length, the segment angles (which determine the step length) were different between fallers and non-fallers. Specifically, fallers showed a smaller thigh angle (less hip flexion) but a larger shank angle (less knee flexion) at NPTD compared to non-fallers. The smaller thigh angle could shorten the step length, while the larger shank angle would enlarge the step length. Hence, both these changes in segment coordination would have a limited effect on the step length, indicating that fallers and non-fallers use different kinematic synergies (segment coordination) to achieve similar gait performance [42]. The usage of kinematic synergies with less joint flexion in fallers might be due to impaired joint motor function post-stroke. People with different levels of impairment or types of joint impairments (hip, knee, or ankle) might make different adjustments in their kinematic synergies. These adjustments could maintain gait performance but might increase the risk of falls, such as the synergies recruited by the fallers.

Previous studies may also have heterogeneous results regarding step length between fallers and non-fallers because they did not consider different fall types [35–37]. We found that the average gait parameters were different between fall types. Trip-fallers showed a shorter step length at PTD compared to the non-fallers, whereas the slip-fallers had a similar step length compared to non-fallers. Therefore, the previous studies which examined spatio-temporal gait parameters for all types of fallers might be affected by the rate of each fall type. For example, a study might demonstrate a significant difference in step length between fallers and non-fallers when a majority of the participants experienced trip-induced falls, while no difference in step length would be found if participants experienced more slip-induced falls.

Trip and slip perturbations are the major causes of the falls in gait, and difference exists in the mechanisms between these two types of falls. Although both types of falls are induced by a disturbance of the lower limb, the trip perturbation is usually related to a disturbance of the swing limb, leading to a forward balance loss (COM exceeded the anterior edge of BOS) [43,44]. Hence, a quick compensatory step is typically required to recover balance loss occurring in the forward direction. Stroke survivors experience motor impairment due to weak ankle dorsiflexor torque seen in the form of a foot drop, which could lead to a lower toe clearance of the swing limb and thus increasing the likelihood of obstacle hit and the risk of trip-related balance loss [45]. Further, motor impairment affecting ankle and foot muscles could also affect the step initiation and step execution; thus, stroke survivors might fail to take a quick forward step after the trip perturbation for balance recovery, especially for the perturbation in early-swing phase, following which elevating strategies (foot liftoff and travel forward to clear the obstacle) are typically used, resulting in difficulty of recovering from the forward balance loss and greater likelihood of a forward fall [46]. Alternatively, the slip perturbation is mostly related to a disturbance of the stance limb, leading to a backward balance loss [43,44]. For backward balance loss recovery from gait-slips, a compensatory forward step or aborted step (loading of the recovery foot without complete toe clearance) from the trailing limb is required. Thus, even if motor impairment affects this compensatory step response, it results in a shorter step length of this step. A shorter or an aborted compensatory step for gait slip is beneficial rather than detrimental for slips, and these strategies lengthen the BOS and increase COM stability [47,48]. Therefore, the trip-fallers could be more affected by the abnormal gait pattern than the slip-fallers. Consistently, our results indicated that the trip-fallers had shorter swing time, smaller thigh angle, larger shank angle, and larger foot angle compared to non-fallers on average, while the slip-fall group only had smaller trunk angle and smaller thigh angle compared to the non-fallers.

Among all the gait kinematics associated with trip-induced fall risk, the thigh angle was smaller at both paretic foot touchdown and non-paretic foot touchdown compared to the non-fallers (*p* < 0.001 for both sides). These results suggest that the control of thigh segment might be one of the key factors affecting the risk of trip-induced fall. A larger thigh angle could lead to a higher toe clearance, thereby lowering the likelihood of obstacle-induced trip perturbation [49,50]. Decreased swing time was also observed in the trip-fall group and could be related to the reduction in thigh angle. Regarding the kinematic measures associated with slip-induced fall risk, the fallers showed a larger trunk flexion compared to the non-fallers, which is inconsistent with previous findings that larger trunk flexion could shift the center of mass (COM) forward and lower the risk of slip-induced fall. It is possible that increased trunk flexion in PwCS might be a compensatory strategy to enhance stability given the decline of knee and hip extensor strength [51]. Therefore, the fallers with larger trunk flexion in gait might also have decreased knee and hip extensor moments, which could affect the propulsive impulse provided by the recovery limb for regaining COM stability after a slip, leading to a higher risk of slip-induced falls [52]. It should be noted these key factors related to fall risk were not detected in all the fallers (Figures 2 and 3), and such findings suggest that the fall risk was not determined by a single factor but by the synergy/tradeoff of multiple factors. For example, a smaller thigh angle (less hip flexion) could lower the toe clearance, increasing the risk of trip-related falls, and a larger shank angle (less knee flexion) could also lower the toe clearance and further increase the trip-related fall risk, while a larger shank angle could increase the toe clearance and compensate the effect of thigh angle on fall risk. Therefore, trip-falls might be due to smaller thigh angle, or larger shank angle, or both for individuals, and these individual differences in fall causes would lead to the relatively smaller amount of group difference in the factors. Even though our results still revealed the common factors affecting fall risk, future study should take into account the synergy and tradeoff among these factors to further validate our findings.

Compared to the fallers, non-fallers showed higher symmetry of trunk angle (SR: 0.99 vs. 1.03) and higher symmetry of foot angle (SI: 0.05 vs. 0.08). Trunk control can help maintain the position of the COM [53,54], and impaired trunk control could thus lead to both postural instability and dynamic instability [55,56]. Therefore, the asymmetrical trunk motion could increase the COM instability, thereby increasing the risk of falls. Regarding the asymmetry in foot angle, it was found that the symmetry index of feet was a significant predictor of walking ability (household or community walker), and stroke survivors with asymmetrical foot posture have more limited walking ability. Therefore, the asymmetrical foot posture might also affect balance control as balance ability was proved to be correlated with walking ability [57]. We found foot angle asymmetry in both slip- and trip-related fallers, while trunk angle asymmetry was only found in trip-related fallers, indicating that the asymmetrical foot posture is a common factor affecting both slip- and trip-induced falls. The foot angle in Table 2 indicated that the higher SI in the trip-fall group was due to larger foot angle (less dorsiflexion) at PTD compared to the angle at NPTD. The less dorsiflexion in the paretic side could reduce the toe clearance during swing phase, leading to a higher risk of trip-fall. Hence, a better control of foot angles might help to reduce the fall risk in PwCS. A few interventions have been reported to improve gait symmetry [58,59], which might also reduce the likelihood of falls in PwCS. It should be noted that the significant difference in SR could not guarantee a significant difference in SI and vice versa. SR was calculated as the ratio of the values for the two limbs, which takes into account the direction of the difference between the two limbs, while SI was calculated as the absolute difference between the values for the two limbs. Therefore, the results were not always consistent between SR and SI.

Some limitations exist in the present study. First, only the kinematic measures in the AP direction were analyzed in this study. We chose these measures because slips and trips were the most frequent cause of falls in our study, which mainly induce a balance loss in the forward or backward direction with recovery movements occurring mainly in the sagittal plane. Additionally, during the task of walking, the joint excursions in the medio-lateral direction are small. However, previous studies have proposed that medial–lateral kinematic measures can also affect fall risk to some extent, such as step-width and sway distance [17,60]; hence, our future study will include more variables in the medial–lateral direction. Second, we used 12-month retrospective fall history to classify fallers and non-fallers, rather than prospective fall information. Thus, it is possible that past fall events could have led to an altered gait pattern collected in our laboratory. Therefore, prospective fall information will be used to further verify the findings in this study. Last, the sample size of 36 could only yield a power >70% and moderate-to-high effect size (0.5 to 0.8) for the key factors with significant difference (Table 4) using a two-way ANOVA analysis. Therefore, the interpretation of our results was limited by the small sample size, and future studies are needed with larger samples to validate these findings.

## 5. Conclusions

In summary, this study compared the average gait kinematics and their symmetry between fallers and non-fallers in people with chronic stroke, and the fallers were classified using 12-month retrospective fall data. It was found that fallers showed a different gait pattern compared to non-fallers, and different fall types were associated with different gait characteristics. Among all the kinematic measures, a common factor affecting both slip and trip fall risk was higher asymmetry of foot angle, indicating that training programs improving foot symmetry might be effective for reducing fall risk. In addition, thigh angle at foot touchdown might be one the key factors affecting fall risk, especially for trip-induced falls, suggesting that stroke patients at high risk of trip-induced falls might benefit from hip-flexor training. Our findings would be helpful to improve the precision of fall-risk prediction models in stroke survivors and design effective fall risk prevention paradigms which can be tailored based on fall risk type. Implementation of these fall risk assessment and prevention paradigms has the potential to reduce fall-related injuries in people with chronic stroke, leading to a reduction in associated hospital costs.

## Figures and Tables

**Table 1.** The mean and SD of anthropometric measures for fall group and non-fall group.

Variable	Fall *(n* = 21)	Non-Fall (*n* = 15)	*p*-Value
Height (cm)	173.3 (10.1)	169.3 (11)	0.27
Weight (kg)	82.4 (16.4)	74.9 (12.3)	0.15
Age (years)	58.1 (7.8)	60.3 (12.3)	0.52
Gender	14 males	9 males	0.69
Foot length (cm)	30.2 (2.3)	28.8 (2.8)	0.11
Foot-to-hip height (cm)	87.3 (5.1)	84.4 (4.6)	0.09
Leg length (cm)	93.6 (5.1)	90.9 (5)	0.14
Femur length (cm)	47.6 (3.8)	46.5 (2.7)	0.38

**Table 2.** The mean and SD of gait kinematics for fall group, slip-fall group, trip-fall group, and non-fall group.

Variables	Non-Paretic Side			
	All-Fall (*n* = 21)	Slip-Fall (*n* = 11)	Trip-Fall (*n* = 7)	Non-Fall (*n* = 15)
Step length/BH	0.26 (0.07)	0.25 (0.08)	0.24 (0.07)	0.27 (0.09)
**Stride length/BH**	0.54 (0.16)	0.53 (0.18)	**0.46 (0.16)** *	0.57 (0.18)
Stance time (s)	117.09 (20.03)	117.5 (23.33)	119.5 (24.22)	121.88 (41.24)
Swing time (s)	43.59 (11.66)	44.76 (13.49)	40.81 (10.75)	46.16 (10.75)
**Trunk (deg)**	**85.65 (5.36)** **	**85.7 (5.42)** *	86.17 (7.04)	88.59 (4.36)
**Thigh (deg)**	**108.33 (5.68)** **	109.71 (6.79)	**103.24 (4.79)** ***	111.72 (4.88)
**Shank (deg)**	**102.12 (7.81)** *	100.53 (7.02)	**103.18 (6.34)** *	98.42 (7.56)
Foot (deg)	75.84 (8.88)	76.82 (9.26)	75.59 (7.63)	73.65 (8.02)
COMx	0.4 (0.06)	0.39 (0.07)	0.39 (0.06)	0.41 (0.04)
Paretic side				
**Step length/BH**	0.27 (0.08)	0.27 (0.1)	**0.22 (0.08)** *	0.27 (0.08)
**Stride length/BH**	0.53 (0.14)	0.52 (0.16)	**0.46 (0.13)** *	0.56 (0.17)
Stance time (s)	103.49 (26.28)	107.24 (34.16)	108.62 (37.51)	110.09 (31.1)
**Swing time (s)**	55.37 (12.44)	53.39 (13.73)	**48.48 (12.93)** **	59.51 (14.75)
Trunk (deg)	88.28 (4.96)	86.68 (4.99)	89.09 (5.27)	88.12 (7.42)
**Thigh (deg)**	**106.4 (4.99)** **	**107.04 (5.63)** *	**103.52 (5.3)** ***	109.43 (4.43)
Shank (deg)	101.77 (6.58)	102.01 (6.45)	99.97 (7.09)	99.87 (9.24)
**Foot (deg)**	75.88 (7.89)	74.91 (8.94)	**78.58 (8.02)** *	73.93 (8.75)
COMx	0.37 (0.04)	0.37 (0.05)	0.36 (0.04)	0.38 (0.05)

Here BH denotes body height, * indicates *p* < 0.05, ** indicates *p* < 0.01, and *** indicates *p* < 0.001.

**Table 3.** The mean and SD of gait asymmetries (SR and SI) for fall group, slip-fall group, trip-fall group, and non-fall group.

Variables	Symmetry Ratio			
	All Fall (*n* = 21)	Slip-Fall (*n* = 11)	Trip-Fall (*n* = 7)	Non-Fall (*n* = 15)
Step length	1.13 (0.51)	1.21 (0.67)	0.96 (0.37)	1.04 (0.27)
Stride length	1.01 (0.18)	1.02 (0.23)	1.06 (0.28)	1.01 (0.11)
Stance time	0.88 (0.13)	0.91 (0.16)	0.89 (0.13)	0.93 (0.14)
Swing time	1.36 (0.45)	1.3 (0.52)	1.27 (0.48)	1.41 (0.68)
**Trunk**	**1.03 (0.06)** **	1.01 (0.06)	**1.04 (0.06)** **	0.99 (0.06)
Thigh	0.98 (0.05)	0.98 (0.05)	1 (0.05)	0.98 (0.05)
**Shank**	1 (0.08)	1.02 (0.07)	**0.97 (0.09)** *	1.02 (0.07)
Foot	1.01 (0.13)	0.98 (0.14)	1.05 (0.15)	1.01 (0.08)
COMx	0.94 (±0.21)	0.97 (0.26)	0.95 (0.22)	0.93 (0.1)
Symmetry Index				
**Step length**	0.25 (0.26)	**0.32 (0.33)** *	**0.3 (0.32)** *	0.17 (0.16)
Stride length	0.08 (0.12)	0.1 (0.15)	0.12 (0.18)	0.07 (0.08)
Stance time	0.17 (0.1)	0.17 (0.1)	0.16 (0.09)	0.14 (0.09)
Swing time	0.35 (0.24)	0.36 (0.26)	0.35 (0.24)	0.36 (0.28)
Trunk	0.05 (0.04)	0.05 (0.04)	0.05 (0.04)	0.05 (0.04)
Thigh	0.04 (0.03)	0.04 (0.04)	0.04 (0.03)	0.04 (0.03)
Shank	0.06 (0.04)	0.06 (0.05)	0.08 (0.06)	0.05 (0.04)
**Foot**	**0.1 (0.08)** **	**0.12 (0.08)** ***	**0.13 (0.06)** ***	0.06 (0.05)
**COMx**	**0.18 (0.13)** ***	**0.21 (0.13)** ***	**0.18 (0.12)** **	0.1 (0.09)

Here * indicates *p* < 0.05, ** indicates *p* < 0.01, and *** indicates *p* < 0.001.

**Table 4.** The results of power analysis for the key factors affecting fall risk. The effect size (Cohen’s d value) and power was calculated for the current sample size (*n* = 36), and the sample size required to reach 80% power was also estimated.

Variable	Actual Effect Size	Actual Power	Sample Size Required > 80% Power
Trunk at NPTD	0.65	74%	42
Thigh at NPTD	0.67	76%	40
Thigh at PTD	0.67	77%	39
SR for Trunk	0.66	76%	40
SI for Foot	0.63	72%	44
SI for COMx	0.72	83%	34

## Data Availability

The data presented in this study are available on request from the corresponding author.
